# Identification of Novel Human NK Cell Progenitor Subsets

**DOI:** 10.3390/ijms18122716

**Published:** 2017-12-15

**Authors:** Priyanka Sathe, Swee Heng Milon Pang, Rebecca Delconte, Ngaire Elwood, Nicholas D. Huntington

**Affiliations:** 1 The Walter and Eliza Hall Institute of Medical Research, 1G Royal Parade, Parkville, VIC 3052, Australia; sathe@wehi.edu.au (P.S.); pang@wehi.edu.au (S.H.M.P.); delconte.r@wehi.edu.au (R.D.); 2 Department of Medical Biology, University of Melbourne, Parkville, VIC 3050, Australia; 3 Cord Blood Research, Murdoch Children’s Research Institute, Melbourne, VIC 3052, Australia; ngaire.elwood@mcri.edu.au; 4 Department of Paediatrics, University of Melbourne, Melbourne, VIC 3010, Australia

**Keywords:** human haematopoiesis, lymphopoiesis, NK cell progenitors

## Abstract

Understanding the pathways and regulation of human haematopoiesis, in particular, lymphopoiesis, is vital to manipulation of these processes for therapeutic purposes. However, although haematopoiesis has been extensively characterised in mice, translation of these findings to human biology remains rudimentary. Here, we describe the isolation of three progenitor subsets from human foetal bone marrow that represent differential stages of commitment to the natural killer (NK) cell lineage based on IL-15 responsiveness. We identify CD7 as a marker of IL-15 responsive progenitors in human bone marrow and find that this expression is maintained throughout commitment and maturation. Within the CD7^+^ fraction, we focussed on the lineage potential of three subsets based on CD127 and CD117 expression and observed restricted lymphoid and biased NK cell potential amongst subsets. We further demonstrate the presence of subsets similar in both phenotype and function in umbilical cord blood and the bone marrow of humanised mice, validating these as appropriate sources of progenitors for the investigation of human haematopoiesis. Overall, we describe several stages in the process of lymphopoiesis that will form the basis of investigating the regulators of this process in humans.

## 1. Introduction

Despite extensive progress in our understanding of the pathways and regulation of murine haematopoiesis, our knowledge of these processes in humans remains undeveloped. In particular, while the myeloid differentiation pathway has been described [1,2] the intermediate stages in the process of lymphopoiesis are less clear. Understanding these intermediate stages is a critical precursor to elucidating the regulators of lymphopoiesis in humans, information that would help better understand immunodeficiencies and becomes increasingly important as more therapies seek to manipulate the numbers or function of lymphoid cells, in particularly natural killer (NK) cells in cancer.

Human common lymphoid progenitors (CLP) have been variously described as CD7^+^, CD10^+^ or CXCR4^+^, with varying levels of CD117 and CD127 expression having been reported [3,4,5,6]. Further downstream, progenitors with T and NK cell potential have been described [7]. Neither B nor myeloid cell potential was detected, although it is unclear whether the assay used, foetal thymic organ culture, was appropriate for detection of these lineages. Whether such a T/NK cell committed progenitor exists remains to be determined.

Recently, Renoux et al. identified a downstream NK cell-restricted progenitor within the Lin^−^CD34^+^CD7^+^CD10^+^ fraction as a human equivalent of the murine NK cell progenitor (NKP) [8]. Both lymphoid primed multipotent progenitors (LMPP) and “CLP-like” cells gave rise to these progenitors in vitro, suggesting the NKP described is a stage in the physiological process of NK cell development.

To track these intermediate stages in which loss of myeloid, T and B cell potential and thus commitment to the NK cell lineage occurs, we have focused on the only factor known to be essential for human and murine NK cell development, IL-15 [9,10,11,12], sensitivity to which marks murine NK cell lineage commitment [13,14,15].

Here, we track in vitro IL-15 responsive progenitors to identify progenitors with NK potential. We characterise the progeny of IL-15 responsive CD34^+^ progenitors and validate the role of these intermediate progenitors in the pathway to NK cell differentiation, including characterisation of their potential for alternate lineages. Further, we have characterised progenitors from a variety of sources of human tissue, finding their lineage potential broadly conserved and validating a new model of humanised immune system mice for studying lymphopoiesis.

Overall, we describe multiple populations of IL-15 responsive progenitors, traceable by uniform expression of the T and NK cell marker CD7, and differential expression of CD117, a marker whose downregulation represents progression into terminal differentiation. Further, we describe conservation of these stages of commitment across different sources of human progenitors. We expect these findings will provide a framework for future studies investigating the regulation of human lymphopoiesis.

## 2. Results

### 2.1. CD7 Identifies the IL-15 Responsive Population Amongst CD34^+^ Progenitors

**Figure 1:**
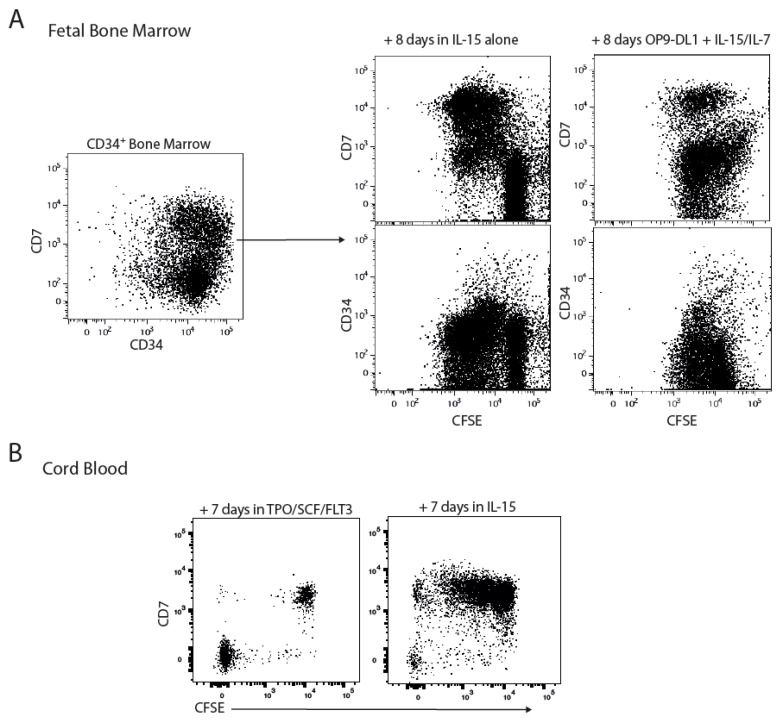
(**A**) CD7^+^ progenitors respond to IL-15. CD34^+^ progenitors purified from human foetal liver were labelled with carboxyfluorescein succinimidyl ester (CFSE) and cultured for 8 days in IL-15 alone or IL-15 + IL-7 + Notch-ligand. FACS plots are representative of 2 experiments. (**B**) Umbilical cord blood cells were isolated and labelled with cell trace violet (CTV) and cultured in either FLT3L + SCF + TPO or IL-15 alone for 7 days. Cultures were analysed for expression of CD7 and CTV. FACS plots are representative of three experiments.

We previously reported an expansion of mature CD7^+^CD56^+^ NK cells in the spleen and also noted a robust expansion of lineage^−^ cells in the bone marrow (BM) of human immune system mice following treatment with IL-15 receptor agonists [11]. The CD7^+^ population in human cord blood and foetal liver has previously been postulated to represent a heterogeneous mix of progenitors, including the common lymphoid progenitor. NK cells develop from CD34^+^CD38^+^ progenitors from various sources after several weeks in stem cell factor (SCF) and IL-15 in vitro [16]. CD7 is expressed on a subset of lin^−^CD34^+^ progenitors in humans thus we purified total CD34^+^ from foetal bone marrow and monitored CD7, CD34 expression amongst the IL-15 responsive cells in vitro. In response to IL-15 alone, only the IL-15 responsive cells (as determined by cell proliferation/dilution of carboxyfluorescein succinimidyl ester (CFSE) expressed CD7 after 8 days of culture (Figure 1A) indicating that either only the CD34^+^CD7^+^ population responds to IL-15 or IL-15 induces CD7 on responsive progenitors. In contrast, in vitro culture of identical progenitors in IL-7 + IL-15 and Notch-ligand resulted in the proliferation of both CD7^+^ and CD7^−^ progenitors, arguing against IL-15 purely inducing CD7 on responsive progenitors (Figure 1A). Consistent with a recent report [17], Notch signalling and IL-7 appear to impair commitment towards the NK cell lineage as evidenced by reduction in proliferating CD7^+^ progenitors. We next assessed if CD7^+^ progenitors derived from cord blood conserved this preferential sensitivity to IL-15. Since lin^−^CD34^+^ progenitors are rare, we assessed the ability of total lin^−^ cord blood cells to respond to IL-15 or thrombopoetin, fms-like tyrosine kinase-3 ligand and SCF (TPO/FLT3L/SCF). While CD7^+^ cells did not proliferate in response to TPO/FLT3L/SCF, CD7^−^ cells proliferated extensively. Consistent with data from foetal bone marrow, we observed an equally biased response to IL-15 by CD7^+^ cells as primarily CD7^+^ cells survived and proliferated after 7 days of culture (Figure 1B). A small subset of CD7^−^ progenitors was also found to proliferate in these cultures. Whether this reflects a separate IL-15 responsive pathway or a population of early progenitors which have yet to acquire CD7 will be an interesting subject for further investigation. This period of in vitro cultivation was not sufficient to induce NK cell or T cell lineage markers (CD56, NKp46, CD161, CD3; data not shown) suggesting they represent IL-15 responsive progenitors. Taken together, this data confirms that human CD34^+^ cells respond to IL-15 and responsive cells preferentially express CD7, a marker of mature NK and T cells.

### 2.2. The Lin^−^CD7^+^ Fraction Consists of Multiple Subsets Defined by CD117 and CD127

**Figure 2:**
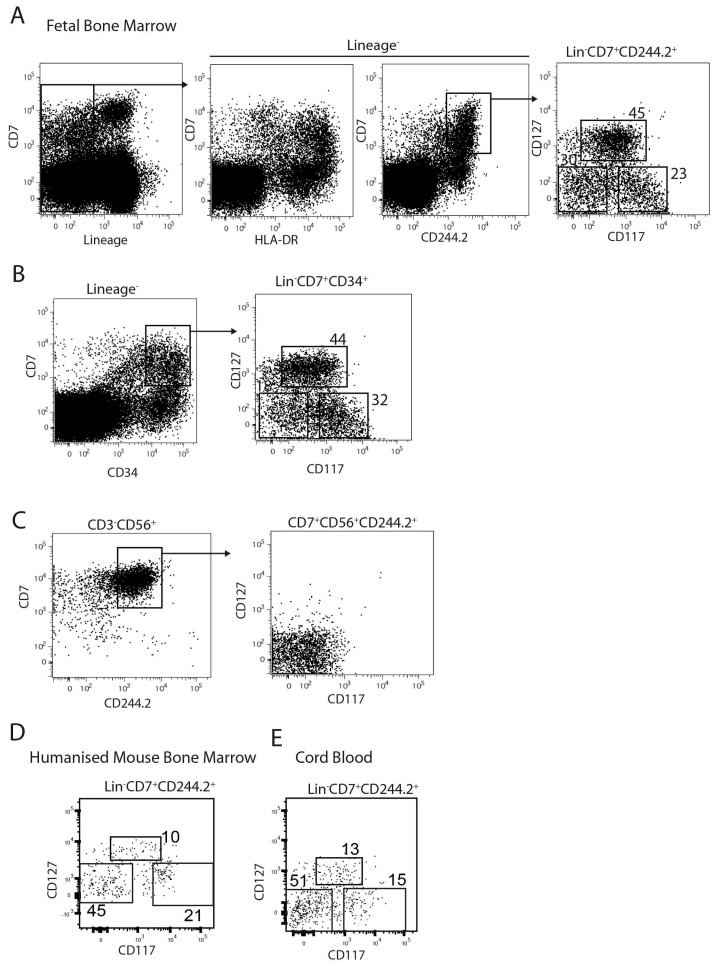
Identification of CD7^+^ progenitor subsets in humans. (**A**) Mononuclear cells were isolated from 13-week foetal bone marrow (BM) and analysed by flow cytometry for expression of lineage markers (CD3, CD56, CD19, CD14) and CD7, HLA-DR, CD244.2 (2B4). Lin^−^CD7^+^CD244.2^+^ progenitors were further characterised as CD117^high^CD127^−^, CD117^int^CD127^+^, CD117^l^^ow^CD127^−^. Percentages are representative of four different donors; (**B**) Mononuclear cells were isolated from 13-week foetal BM and analysed by flow cytometry for expression of lineage markers (CD3, CD56, CD19, CD14) and CD7 and CD34. Lin^−^CD7^+^CD244.2^+^ progenitors were further characterised as CD117^high^CD127^−^, CD117^int^CD127^+^, CD117^l^^ow^CD127^−^. Percentages are representative of four different donors; (**C**) CD56^+^CD3^−^ NK cells from 13-week foetal BM were analysed by flow cytometry for expression of CD7, CD244.2, CD117 and CD127. FACS plots are representative of four donors. Lin^−^CD7^+^CD244.2^+^ progenitors from (**D**) humanised mouse bone marrow or (**E**) umbilical cord blood was analysed for the CD127/CD117 progenitor subsets identified in (**A**). FACS plots are representative of three biological replicates.

We previously reported an expansion of mature CD7^+^CD56^+^ NK cells in the spleen and also noted a robust expansion of CD7^+^CD56^−^ cells in the BM of human immune system (HIS) mice following treatment with IL-15 receptor agonists [11]. The CD7^+^ population in human cord blood and foetal liver has previously been postulated to represent a heterogeneous mix of progenitors, including the common lymphoid progenitor. To determine whether the CD7^+^ IL-15 responsive population in HIS BM was a similar mix of lymphoid progenitors, we further characterised this population based on markers who have previously reported to be differentially expressed on progenitors at differential degrees of lineage restriction; CD117 (c-kit) and CD127 (IL-7Rα). Lineage negative progenitors in human foetal bone marrow contain a CD7^+^ fraction which co-express MHC-II (HLA-DR) and CD244.2 (2B4). Within the Lin^−^CD7^+^CD244.2^+^ population we found three distinct subsets with the Lin^−^CD7^+^ fraction, based on CD117 and CD127 expression; CD117^high^CD127^−^, CD117^int^CD127^+^ and CD117^low^CD127^−^ cells (Figure 2A). Similarly, these three subsets were observed at an almost identical frequency gating on Lin^−^CD7^+^CD34^+^ indicating that Lin^−^CD7^+^HLA-DR^+^CD244.2^+^ CD34^+^ human foetal bone marrow progenitors contain three distinct subsets based on CD127 and CD117 expression (Figure 2B). In contrast, mature NK cells from the foetal bone marrow (CD7^+^CD56^+^CD244.2^+^ CD3^−^) failed to express CD117 nor CD127 (Figure 2C). To investigate whether these IL-15 responsive HIS BM progenitors reflected genuine developmental intermediates, we searched for similar progenitors in foetal BM, the primary site of human lymphopoiesis, and umbilical cord blood (UCB), a commonly used source of human haematopoietic tissue. We found phenotypically identical CD117^high^CD127^−^, CD117^int^CD127^+^ and CD117^low^CD127^−^ as well as CD117^low^CD127^+^ progenitors within the Lin^−^CD7^+^ CD244.2^+^ fraction of both humanised mouse bone marrow (Figure 2D) and cord blood (Figure 2E).

### 2.3. Differential Lineage Potential of Progenitors

**Figure 3:**
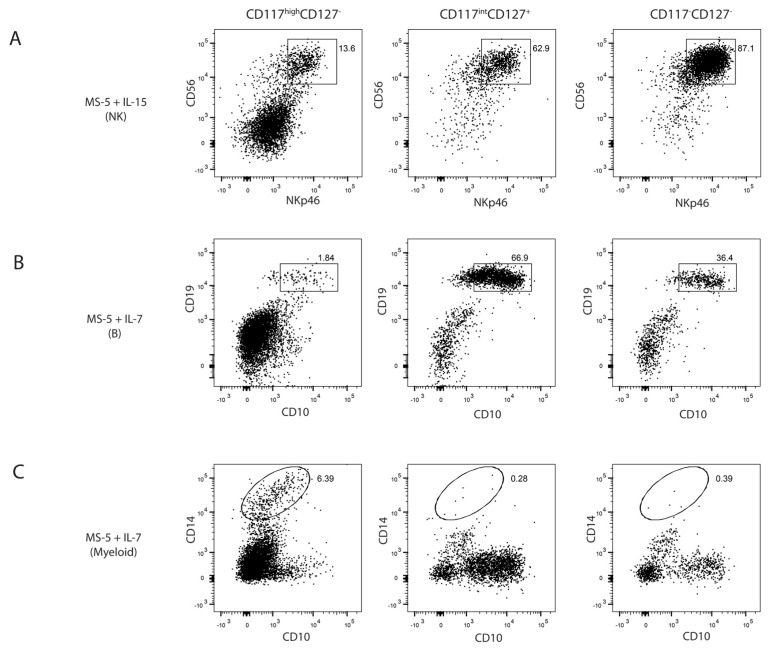
Lineage potential of foetal BM progenitors. Lin^−^CD7^+^ progenitors were sorted from foetal BM as CD117^high^CD127^−^, CD117^int^CD127^+^ or CD117^low^CD127^−^. (**A**) Progenitors were cultured on MS-5 stromal cells with IL-15 and analysed after 7 days of culture for the presence of CD56^+^NKp46^+^ NK cells; (**B**,**C**) Progenitors were cultured on MS-5 stromal cells with IL-7 and analysed after 7 days of culture for the presence of (**B**) CD10^+^CD19^+^ B cells and (**C**) CD10^−^CD14^+^ monocytes and macrophages. FACS plots are representative of 2 experiments.

We had identified IL-15 responsive cells that could be found in multiple sources of human tissue. To determine whether these were indeed NK cell progenitors, and more broadly, the developmental potential of these cells, we sorted each fraction from human foetal BM, as this is the physiological site of human lymphopoiesis, and tested their lineage potential both in vivo and in vitro. All three fractions efficiently gave rise to CD56^+^NKp46^+^ NK cells in vitro as expected (Figure 3A). We were unable to observe T cell differentiation in these in vitro assays while all subsets gave rise to B cells (Figure 3B)**.** Interestingly, despite their expression of CD7, long postulated to be a marker of lymphoid restriction, the CD117^high^CD127^−^ fraction efficiently generated CD14^+^ monocytes (Figure 3C). Neither the CD117^int^ or CD117^low^ progenitors retained this myeloid potential, consistent with a model in which, as in the murine system, decreasing levels of CD117 denoted increasing lineage restriction.

### 2.4. CD7^+^ Progenitor Subset Potential in HIS BM and Cord Blood

**Figure 4:**
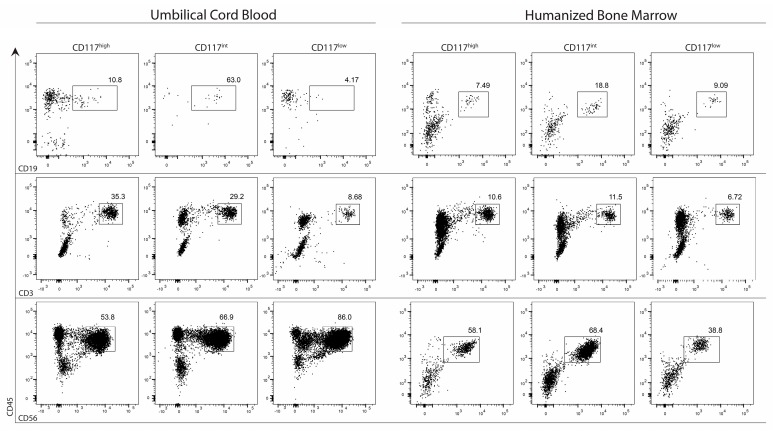
Lineage potential of HIS BM and umbilical cord blood (UCB) progenitors. Lineage^−^CD7^+^ progenitors were sorted from HIS BM or UCB as CD117^high^CD127^−^, CD117^int^CD127^+^ or CD117^l^^ow^CD127^−^ as indicated. Progenitors were cultured on OP9 stromal cells with IL-7 or IL-15 and analysed after 14 days for the presence of CD45^+^CD19^+^ B cells or CD45^+^CD56^+^ NK cells respectively or on OP9-DL1 stromal cells with IL-7 and analysed after 21 days for the presence of CD45^+^CD3^+^ T cells. FACS plots are representative of 2 experiments.

We had found subsets within the Lin^−^CD7^+^ population that displayed progressive lineage restriction, suggesting a temporal progression from the CD117^high^ population to the CD117^low^ fraction in the process of human lymphopoiesis. Given the inherent difficulty in obtaining human foetal tissue, and the presence of phenotypically similar progenitors in models of human lymphopoiesis, including umbilical cord blood (UCB) and our newly described HIS BM, we wanted to determine whether this process of lineage restriction was conserved in the UCB and HIS BM CD117^high^CD127^−^, CD117^int^CD127^+^ and CD117^low^CD127^−^ progenitors. We therefore sorted each fraction from cord blood or HIS BM (Figure 4), and analysed their responses to various cytokine stimuli. We found that no population from either cord blood or HIS BM gave rise to any progeny in response to granulocyte macrophage colony stimulating factor (GM-CSF) [18], suggesting a lack of myeloid potential in any of these progenitors. This discrepancy not withstanding, cord blood or HIS BM derived progenitors followed the same broad process of lymphoid lineage commitment as their foetal bone marrow counterparts. Both the CD117^high^CD127^−^ and CD117^int^CD127^+^ fractions efficiently generated B cells, T cell and NK cells, with B cell potential peaking (by proportion of progeny) in the CD117^int^CD127^−^ population. 

### 2.5. Frequency of Lymphoid Progenitors within CD7 Populations

We had demonstrated a broadly similar pattern of lineage commitment in the Lin^−^CD7^+^ fraction that could be tracked by differential expression of CD117 and CD127. However, from these assays, it was unclear whether these were homogeneous populations with the potentials seen in bulk assays, or whether a subset of each population was giving rise to the lineages we observed in vitro and in vivo. To determine the degree of heterogeneity within these populations, thus better characterising the lymphoid progenitors, we first sought to quantify the frequency of progenitors with potential for each lymphoid lineage. Since our bulk culture assays had shown HIS BM to closely reflect the lineage potential of foetal BM fractions, we analysed the CD117^high^, CD117^int^ and CD117^low^ fractions from HIS BM in limit dilution assays under the conditions that supported the growth of T cells, NK cells or B cells.

**Figure 5:**
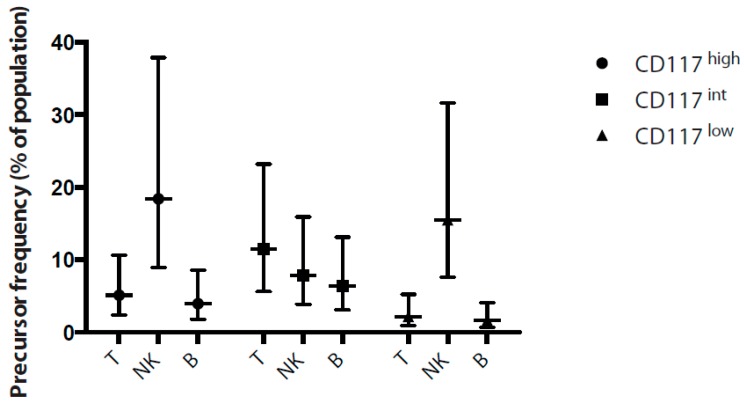
Frequency of lymphoid progenitor potential within lin-CD7^+^ fractions. Lineage^−^CD7^+^ progenitors were sorted from HIS BM as CD117^high^CD127^−^, CD117^int^CD127^+^ or CD117^l^^ow^CD127^−^ as indicated. Varying numbers of progenitors from 27 cells to 1 cell per well were cultured on OP9 stromal cells with IL-7 or IL-15 and analysed after 14 days for the presence of CD45^+^CD19^+^ B cells or CD45^+^CD56^+^ NK cells respectively or cultured on OP9-DL1 stromal cells with IL-7 and analysed after 21 days for the presence of CD45^+^CD3^+^ T cells. Wells with more than 2 cells were scored as positive, and progenitor frequency estimated using Extreme Limiting Dilution Analysis (ELDA) software (https://www.elda.at/portal27/eldaportal/content?contentid=10007.682446&viewmode=content).

We found that the frequency of progenitors with B, T and NK cell potential varied between the subsets (Figure 5). Consistent with the results seen in our bulk assays, the CD117^int^CD127^+^ fraction contained the greatest frequency of B cell progenitors. This population had a B-cell precursor frequency of approximately twice the CD117^high^ fraction, while the CD117^low^ population had few to no B-cell progenitors. Interestingly, T-cell potential seemed to correlate with this trend, while NK progenitor frequency trended inversely to B-cell potential. However, we found a low frequency of progenitors for any lineage amongst all the progenitor fractions, suggesting a high level of heterogeneity in all the progenitor populations. Elucidating the nature of this heterogeneity will require single cell analysis of progenitor potential.

## 3. Discussion

NK cells are short lived innate effector lymphocytes that spontaneously react against transformed and pathogen infected tissues. The peripheral pool of mature NK cells requires constant replenishment from bone marrow progenitors [19]. Upon commitment to the NK cell lineage, NK cell homeostasis is regulated by the growth factor IL-15 [20]. We here describe novel stages in the pathway of early NK cell commitment, prior to expression of conventional NK cell markers and driven by IL-15 responsiveness. Further, we describe conservation of these patterns of lineage commitment, albeit with differential stages of commitment, across multiple sources, validating a humanised immune system (HIS) mouse as a physiological model for lymphopoiesis.

CD7 is a transmembrane protein member of the Ig superfamily with a potential role in NK cell adhesion [21], and expressed highly on mature NK cells. We describe a linear pathway of restriction to the NK cell lineage that can be tracked by CD7 expression and as is the case for the murine NK lineage, increasing IL-15 responsiveness. Intriguingly, contrary to that described in mice, the earliest IL-15 responsive human progenitors are not NK cell restricted, but rather retain both lymphoid and myeloid potential, raising questions around the differential role of IL-15 in NK cell lineage specification. Whether this lineage plasticity has a physiological role, and whether these alternate potentials manifest under physiological conditions, are harnessed under emergency conditions in vivo, or represent residual potential only realised in the presence of supraphysiological levels of cytokines, is unclear. Indeed, in vitro cultivation of highly purified human CD34^+^ progenitors in IL-15 results in the clear proliferation of a fraction of these progenitors without obvious induction of an NK cell commitment program in the proliferating population.

A common source of variability in studies of human haematopoiesis is that range of progenitor sources used, as access to human tissue varies widely. Further, there remains a need for a model that resembles the physiological pathway of human haematopoietic cell generation as in vivo manipulation of the immune system, such as for cancer immunotherapy, becomes increasingly common. We here demonstrate an identical pattern of lineage restriction in HIS BM as that seen in foetal BM, the primary site of human lymphopoiesis, suggesting the haematopoietic lineages that develop in these mice do so along pathways that mirror their physiological counterparts in humans. Thus, we suggest the HIS mouse model as an ideal tool to study the stages of human haematopoiesis.

In limit dilution assays, we found surprisingly low precursor potential amongst all precursor fractions. While this may be in part due to the limitations of the assay in efficiently supporting the development of lymphoid cells from all potential progenitors, it also suggested the progenitor fractions are highly heterogeneous. Traditionally, such heterogeneity has been delineated by the further subsetting of the precursor fractions by surface markers. However, the relative lack of available reagents and known surface markers in humans may preclude such fine subsetting. Given recent advances in genomic technology, we suggest the progenitors we have described here form the basis for isolation of progenitors for single cell RNA sequencing, which would provide further insights into progenitor heterogeneity and regulation. Although such work is beyond the scope of this study, our description of the frequency of progenitors with B, NK and T cell lineage in each fraction and our description of the consistency of progenitor potential across different sources of human haematopoietic tissue lays an exciting foundation for correlating progenitor potential with the gene expression profile of cells within these populations, thus identifying regulators of this process.

## 4. Materials and Methods

### 4.1. Mice

Balb/C immunodeficient mice have been previously described [11,22]. Experimental mice were specific-pathogen-free (SPF) and all animal work was conducted with standard operating procedures approved by The Walter and Eliza Hall Institute of Medical Research animal ethics committees AEC2015.017 (1/3/2015). Mice with human immune systems were generated by irradiating newborn (3–5 day-old) mice with 3 Gy total body irradiation from a γ source, and were injected intra-hepatically (i.h.) with 5 × 10^5^–3× 10^6^ CD34^+^ human cord blood cells obtained from the Bone Marrow Donor Institute/Cord Blood Research Unit (Murdoch Children’s Research Institute, Parkville, VIC, Australia) following approval from The Walter and Eliza Hall Institute of Medical Research Human Research Ethics Committee. 

### 4.2. In Vitro Assays of Progenitor Potential

Progenitors were isolated as indicated and were cultured on either MS-5 or OP9 stromal cells with IL-7 (50 ng/mL) to determine B cell potential; on MS-5 stromal cells with granulocyte macrophage colony stimulating factor (GM-CSF (25 ng/mL) to determine myeloid potential; on either MS-5 or OP9 stromal cells with IL-15 (50 ng/mL) to determine NK cell potential; or on OP9-DLI stromal cells with IL-7 (50 ng/mL) to determine T cell potential. Cells were split 1:2 onto fresh stromal cells and fresh cytokine media if required. Myeloid assays were analysed after 7 days for the presence of CD33^+^CD14^+^ monocytes and CD33^+^CD16^+^ granulocytes; B cell and NK cell assays were analysed after 14 days for the presence of CD45^+^CD19^+^ B cells or CD45^+^CD56^+^ or NKp46^+^ NK cells respectively; and T cell assays analysed at 21 days for the presence of CD45^+^CD3^+^ T cells. All cultures were analysed by flow cytometry as described below. Foetal bone marrow cultures were performed in 48 well plates in DMEM/10% AB serum + IL-15 (50 ng/mL) or on OP9-DL1 with IL-7 (50 ng/mL) and IL-15 (50 ng/mL). Cells were purified as indicated and labelled with 5 μM carboxyfluorescein succinimidyl ester (CFSE) (Molecular Probes, Eugene, OR, USA). 

### 4.3. Flow Cytometry Analysis for Cell-Surface and Intracellular Markers

The following anti-human mAb were used to stain cell suspension for FACS analysis: CD3 (SK7), CD4 (SK3), CD34 (581), CD8 (SK1), CD19 (HIB19), CD10 (HI10a), CD38 (HB7), NKp46 (9E2), CD16 (3G8), CD161 (DX12), CD56 (B159), HLA-DR (L243), CD244.2 (2B4), HLA-A/B/C (G46-2.6), CD117 (YB5.B8), CD14 (M5E2), CD122 (Mik-3), TCR-β (T10B9.1A-31), CD127 (hIL-7R-M21), CD11c (B-ly6), CD7 (M-T701), CD45 (2D1) from (BD Bioscience, CA, USA). All washings and reagent dilutions were done with phosphate buffered saline (PBS) containing 2% foetal calf serum (FCS). All acquisitions were performed using FACSVerse cytometers and cell sorting was perform using FACS ARIA, all machines were interfaced to the FACS-Diva software (BD Bioscience). Data analysis was performed using FlowJo software from TreeStar Inc. (Ashland, OR, USA).

### 4.4. Cell Preparation

Umbilical cord blood was obtained from the Bone Marrow Donor Institute Cord Blood Bank (Murdoch Children’s Research Institute, Parkville, VIC, Australia). Experiments were approved by The Walter & Eliza Hall Institute of Medical Research Human Research Ethics Committee (HREC12/08). Magnetic enrichment of CD34^+^ cells (>98% pure) was performed by using the CD34 Progenitor Cell Isolation Kit (Miltenyi Biotech, Auburn, CA, USA), after preparation of single-cell suspension and isolation of mononuclear cells by density gradient centrifugation over Ficoll-Hypaque (Nycomed Pharma, Roskilde, Denmark). Cell suspensions were prepared in PBS medium with 2% foetal calf serum. Single cell suspensions of murine organs were prepared as previously described [23]. Human foetal bone marrow was a kind gift from Professor Hergen Spits (AMC, Amsterdam-Zuidoost, The Netherlands) and obtained from elective abortions approved by the Medical and Ethical Committee at AMC-UvA. Human NK cells were purified from donor blood buffy coat prepared by density gradient centrifugation over Ficoll-Hypaque (Nycomed Pharma, Zürich, Switzerland) using anti-CD56 magnetic beads (Miltenyi Biotech, Bergisch Gladbach, Germany). Purified cells (CD34^+^) were loaded with 5 µM CFSE (Invitrogen, Carlsbad, CA, USA) or 5 µM cell trace violet (CTV) and cultured at 1 × 10^5^ cells/mL in StemSpan (STEMCELLS Technologies, Vancouver, Canada) with 10% human serum and either 50 ng/mL recombinant human IL-15 (rhIL-15; Peprotech, NJ, USA) or human thrombopoietin (TPO), fms-like tyrosine kinase 3 ligand (FLT3L) and stem cell factor (SCF) (100 ng/mL).

### 4.5. Statistical Analysis

Statistical analyses were performed using GraphPad Prism (GraphPad Software, San Diego, CA, USA). All data were subjected to two-tailed unpaired Student *t* test analysis. Limit dilution assays were analysed using extreme limit dilution assay (ELDA) software (Hu and Smyth, 2009).
